# Unveiling blood donation knowledge, attitude, and practices among 12,606 university students: a cross-sectional study across 16 countries

**DOI:** 10.1038/s41598-024-58284-4

**Published:** 2024-04-08

**Authors:** Nael Kamel Eltewacy, Hossam Tharwat Ali, Tarek A. Owais, Souad Alkanj, EARG Collaborators, Mohamed Elbahnasawy, Arwa Hussein Bilil Ahmed, Mareb H. Ahmed, Amna Liaquat, Ekram Hasanin, Mariam B. Moghari, Munira Dawod Alla jabo, Nidaa T. Alhumaidi, Abdelrhman Muwafaq Janem, Halmat subhi sulaiman, Mohammed Amir Rais, Romana Riyaz, Moath Salem, Moath Ahmed Al-Mekhlafi, Hassan Mumtaz, Ali Osman Balkan, Narjiss Aji, Haya Hammad, Layan Majed Daghash, Mohamed Ebrahim Abdulhusain, Justice Kwadwo Turzin, Firas Aborigiba, Maya Mohamed Ali, Afrah Humaidan Sulaiman, Abdulrahman Allahham, Abdulghani Ahmed Al-Aswadi, Maab Saifaldin Mohammed Alzain, Osama Al Horani, Yosra Hussein Abo El-Azm, Mahmoud Alballa Almahdi, Malak Ramadan Elba, Esraa Mohamed Zedan, Ishmael Yaala, Adnan Alswiti, Zaid Hamdan, Khaled Saifullah, Israa Al-fayyadh, Zainab Khalid Abdulmutalab, Reem Chebli, Hale Betül Gönül, Jaasira Ansari, Zahra Ali Mohamed, Nawal Mahboob Basha, Alina Sami Khan, Fatima Amatur Raheem, Rasha Ashraf Alwredat, Assia Salah, Raneem El-Faouri, Khlood Saleh Al-Ansi, Ahmad Othman, Zainab Ali Shaker Hasan, Albaraa Muad Alshargabi, Musab Bouhajra, Idris Sula, Nasreen ahmad faq ali, Hamza Faida, Meryem Ertuğrul, Hassan Aboul-Ella, Jarjees A. Sulaiman, Nadir Emre Herdan, Soumia Haddoubenderbal, Djedidi Lamis, Emmanuel Boateng Agyenim, Mohammed Abdul Kabir, Qassim Ali, Mostafa Barakat, Shehab Mahdi AL-Ariqi, Eman Fayez Aljazzar, Fatema Abdulwahed Hasan, Kelvin Yeboah, Sarah Saleh Mohamed, Sahar Elazab Ahmed, Sulemana Mohammed, Abubakar Nazir, Abrar AbuHamdia, Joyous Ocran, Manar Hasan, Ikram Khabab, Mohamed Mostafa Mohamed, Ateeba Kamran, Belmegharbi Rania, Abdulrhman Alkhaled, Mohammad Hasan, Mahmoud A. Ebada

**Affiliations:** 1 École Normale Supérieure Paris-Saclay, 91190 Gif-sur-Yvette, France; 2 Eltewacy Arab Research Group (EARG), Cairo, Egypt; 3 Qena Faculty of Medicine, South Valley University, Qena, Egypt; 4 Faculty of Pharmacy, Beni-Suef University, Beni-Suef, Egypt; 5 Faculty of Medicine, Zagazig University, Zagazig, El-Sharkia Egypt; 6 Egyptian Fellowship of Neurology, Ministry of Health and Population of Egypt, Cairo, Egypt; 7 Emergency Medicine and Traumatology Department, Faculty of Medicine, Tanta University, Tanta, Egypt; 8 Karary University, Omdurman, Sudan; 9 Alnoor University College, Mosul, Iraq; 10 Central Park Medical College, Lahore, Pakistan; 11 Faculty of Medicine, University of Tripoli, Tripoli, Libya; 12 Al-Azhar University Gaza (AUG), Gaza, Palestine; 13 Alzaiem Alazhari University (AAU), Khartoum, Sudan; 14 Faculty of Pharmacy, Taif University, Taif, Saudi Arabia; 15 Faculty of Medicine, Al Quds University, Jerusalem, Palestine; 16 College of Medicine, University of Duhok, Duhok, Iraq; 17 Faculty of Medicine of Algiers1, University of Algiers1, Algiers, Algeria; 18 Department of General Medicine, Shadan Institute of Medical Sciences, Hyderabad, Telangana India; 19 Faculty of Medicine, Damascus University, Damascus, Syria; 20 Faculty of Medicine, Thamar University, Dhamar, Yemen; 21 Maroof International Hospital, Islamabad, Pakistan; 22 Faculty of Medicine, Bezmialem University, Istanbul, Turkey; 23 Faculty of Medicine and Pharmacy of Rabat, Mohammed V University, Rabat, Morocco; 24 Islamic University of Gaza, Gaza, Palestine; 25 The Hashemite University, Zarqa, Jordan; 26 Kasr Alainy Medical School, Cairo University, Giza, Egypt; 27 Department of Biomedical Science, School of Allied Health Sciences, University of Cape Coast, Cape Coast, Ghana; 28 Faculty of Medicine, Aleppo University, Aleppo, Syria; 29 Faculty of Medicine, University of Khartoum, Khartoum, Sudan; 30 College of Medicine, Sulaiman Alrajhi University, Albukayriah, Al-Qassim Saudi Arabia; 31 Faculty of Medicine and Health Sciences, Sana’a University, Sanaa, Yemen; 32 Stemosis Association for Scientific Research, Damascus, Syria; 33 Faculty of Medical Technology, University of Tripoli, Tripoli, Libya; 34 Faculty of Medicine, Al-Azhar University in Cairo, Cairo, Egypt; 35 University of Jordan, Amman, Jordan; 36 Almughtaribeen University, Khartoum, Sudan; 37 University of Mohammed V of Health Sciences, Rabat, Morocco; 38 Liaquat National Hospital and Medical College, Karachi, Pakistan; 39 Hashemite University, Zarqa, Jordan; 40 Faculty of Medicine, Algiers University, Algiers, Algeria; 41 General Surgery Department, Hashemite Universit, Zarqa, Jordan; 42 21 September University of Medical and Applied Science, Boston, USA; 43 Faculty of Medicine, MMMP, Mansoura University, Mansoura, Egypt; 44 Faculty of Medicine and Pharmacy of Rabat, Rabat, Morocco; 45 College of Applied Sciences, Sulaiman Al Rajhi University, Bukayriah, Al Qassim Saudi Arabia; 46 College of Medicine, University of Zakho, Zakho, Iraq; 47 Medical School, İstanbul University, Istanbul, Turkey; 48 Faculty of Veterinary Medicine, Cairo University, Giza, Egypt; 49 Bezmialem Vakif University, Istanbul, Turkey; 50 University Abu Bekr Belkaid—TLEMCEN, Tlemcen, Algeria; 51 Faculty of Medecine, University of Constantine 3, El Khroub, Algeria; 52 University of Cape Coast, Cape Coast, Ghana; 53 University of Science and Technology, Yemen (UST), Aden, Yemen; 54 Faculty of Medicine, Islamic University of Gaza, Gaza, Palestine; 55 Faculty of Public Relations and Media, University of Tripoli, Tripoli, Libya; 56 Faculty of Pharmacy, South Valley University, Qena, Egypt; 57 Department of Biomedical Science, The University of Cape Coast, Cape Coast, Ghana; 58 King Edward Medical University, Lahore, Pakistan; 59 Department of Medical Laboratory Science, Faculty of Medicine and Health Science, An-Najah National University, Nablus, Palestine; 60 King Hamad University Hospital, Al Sayh, Bahrain; 61 Faculty of Medicine, University of Badji Mokhtar Annaba, Annaba, Algeria; 62 Karachi Medical and Dental College, Karachi, Pakistan; 63 Annaba, Algeria; 64 Faculty of Dentistry, Tishreen University, Lattakia, Syria; 65 Aga Khan University Hospital, Nairobi, Kenya

**Keywords:** Blood donation, University students, Multinational, Cross-sectional study, Health care, Medical research, Cancer

## Abstract

We assessed university students’ knowledge, attitude, and practice toward blood donation and identified the factors that promote or hinder their willingness to donate. We employed a multicenter cross-sectional design, collecting data from August to October 2022 through self-administered questionnaires available in Arabic and English. Both online (Google Forms) and paper surveys were utilized. Data were analyzed using R Statistical Software (v4.1.3; R Core Team 2022). A total of 12,606 university students (7966 females and 4640 males) from 16 countries completed the questionnaire; of them, 28.5% had a good knowledge level regarding blood donation, and 22.7% had donated blood at least once. Students in health science colleges had significantly more awareness of blood donation (*p*-value < 0.001), but there were no significant differences in practice (*p*-value = 0.8). Barriers to donation included not being asked (37%), medical ineligibility (33%), fear of pain or infection (18%), concerns about negative health effects (18%), difficulty accessing donation centers (15%), and medical mistrust (14%). Individuals aged > 20 years had significantly higher odds of possessing a high knowledge level (adjusted odds ratio [aOR] 1.77, *p* < 0.001). Private and international university enrollment was associated with increased knowledge (aOR 1.19, *p*-value < 0.001 and aOR 1.44, *p*-value = 0.003), while non-health science college students had lower odds (aOR 0.36, *p* < 0.001). Regarding blood donation status, participants > 20 years old were more likely to donate (aOR 2.21, *p* < 0.001). Conversely, being female, having congenital or chronic diseases, and possessing low knowledge levels were associated with decreased odds of blood donation (all *p* < 0.05). University students show insufficient knowledge about blood donation, with health science students displaying higher awareness levels. Despite their positive attitudes, blood donation rates remain low across all disciplines. It is imperative to enhance education and accessibility to foster a culture of blood donation among students.

## Introduction

Blood is a vital human body component, constantly breaking down and synthesizing through natural processes. Despite remarkable advancements in medicine and technology, artificial synthesis of blood is still impossible, rendering donation the sole means of providing blood and its components^1^. With the rise in life expectancy, traumatic accidents, blood diseases, cancers, and obstetrical complications, blood transfusion has become an essential management approach for numerous life-threatening conditions^2–4^.

Providing sufficient, secure, accessible blood is challenging in developing nations^5^. Every year, over 112 million units of blood are collected, with nearly half of them obtained in high-income nations. Additionally, on average, the donation rate in high-income nations is nine times greater than in low- and middle-income countries (LMICs)^6^. As a result, LMICs have greater blood demands but lack a maintained blood supply^5,7^.

According to the World Health Organization (WHO), Red Cross, and Red Crescent Societies, there are three categories of blood donations: voluntary, replacement, or paid donation^8^. In several countries, the majority of blood is obtained from replacement donors in hospitals, who donate when a friend or family member is in need. Nevertheless, voluntary donation is the most dependable way to fulfill national blood transfusion needs^9^. Donors who voluntarily donate blood once or twice a year are considered the safest because they are not incentivized to provide false information in order to donate^10^.

However, only 62 countries currently have blood supply systems that rely entirely on voluntary non-remunerated donations, according to the World Health Organization (WHO). As a result, blood donation organizations are exploring the potential of incentives or rewards to increase donor recruitment. Research suggests that people may be more likely to take action if they are sufficiently motivated or incentivized^11^.

As the demand for blood donors increases, recruiting them becomes more challenging. It is ideal for sourcing blood donations from young individuals who are healthy, energetic, and have the potential to be long-term prospects^3^. The World Health Organization recommends that at least 1% of the population should donate blood to meet the country’s essential blood requirement, making young adults a significant contributor. However, data shows that young people are the least represented in blood donation^12^. A recent study in Qatar revealed that only 15% of university students were blood donors^13^, while studies in Saudi Arabia reported a prevalence of blood donation among university students ranging from 19 to 45%. However, it was found that most donors only made a single donation and did not regularly donate^8,14–16^. To engage this valuable source, it is crucial to determine their knowledge, motivations, barriers, and behavior toward blood donation^17^.

Consequently, our research endeavors to bridge the knowledge gap about blood donation among university students in our region. Our primary objective is to assess their knowledge level of blood donation and ascertain whether any notable differences exist between students enrolled in health science colleges and those in non-health science colleges. Additionally, we intend to scrutinize any hurdles that could impede or diminish the donation frequency amongst this demographic. Furthermore, we endeavor to identify the incentives that drive university students to participate in blood donation. Lastly, through the dissemination of our findings, we aspire to promote consciousness about the significance of blood donation.

## Materials and methods

### Study design, population, and recruitment procedure

We conducted a multicenter, cross-sectional study in 16 countries (Algeria, Bahrain, Egypt, Ghana, India, Iraq, Jordan, Libya, Morocco, Pakistan, Palestine, Saudi Arabia, Sudan, Syria, Turkey, and Yemen). The study was done between August 2022 and October 2022 using online and/or paper surveys. The study adhered to the Strengthening The Reporting of Observational Studies in Epidemiology (STROBE) Checklist in its entirety^18^. Convenience and snowball sampling methods were used to recruit eligible study participants. The sample size was calculated using Epi Info statistical calculator 7.2.5. version, a trademark of the Center for Disease Control and Prevention (CDC), with the following parameters: a confidence interval of 95%, an expected frequency of 50%, and an acceptable margin of error of 5%. The minimum sample size for each country was 400 responses.

### Eligibility criteria

Male and female university students aged between 18 and 25 years from the selected countries who could respond to the questionnaire in English or Arabic were invited to participate in the study. Ineligible individuals and those who had previously filled in the survey were excluded.

### Study tool

The questionnaire utilized in the study was informed by numerous prior studies conducted globally^2,6,12–14,17,19^. The questionnaire, available in both English and Arabic, was developed as a self-administered Google form survey. To prevent the repetition of responses, the questionnaire was configured to permit only one response per associated email. The questionnaire covered four domains: sociodemographic data, knowledge about blood donation, attitude toward blood donation, and blood donation practices. Sociodemographic data included age, sex, country of residence, the original place of residence, type of university, college, and health status.

The blood donation knowledge section included information about the individual’s blood group, the right to voluntary blood donation, the amount of blood donated at a time, and the health requirements for donation. The blood donation practices section included donation status, intention to donate in the future, and if practiced, the type of donation, frequency, and quantity. The attitudes towards blood donation section included motivating and preventing factors, the role of social media, the influence of individuals on their friends, and the attitudes of university, friends, and family.

### Validation and pilot study

To validate the content of the survey, experts in the hematology and public health field were invited to fill in the survey and assess the clarity, comprehension, and relevance of each question to the measured outcome (knowledge, attitude, or practice). Post validation, a pilot study was conducted on 25–35 participants from each of the 16 countries in the Middle East and North Africa (MENA) region. We employed Cronbach’s alpha to evaluate the reliability and internal consistency of the survey. The internal consistency for the knowledge section was deemed acceptable, with a value of 0.63.

### Data collection

An online link to the Google form was distributed among university students through social media platforms. The link recorded the data anonymously and did not record any contact or personal information. We invited students who lacked internet access or the survey link to participate in the study by completing a paper questionnaire. Subsequently, the study collaborators entered the responses from the paper questionnaires into the study’s database. At the onset of the questionnaire, the participant was presented with the choice to grant or decline study participation. If they opted to participate, they were required to specify their preferred language, either Arabic or English. Following this, we included two confirmatory questions: the first to ascertain the individual’s eligibility to participate and the second to ensure that they had not already completed the questionnaire for the same study, thereby preventing data duplication. Participants with incomplete responses were excluded to prevent any potential information bias.

### Ethical considerations

The study was conducted according to the principles of the Declaration of Helsinki (1964, last revised in 2013)^20^. This survey was voluntary, and participants provided their informed consent by marking a checkbox to signify their willingness to participate in the study. Participants’ anonymity and confidentiality were ensured throughout the study, including data collection and analysis. Initial ethical approval was obtained from the institutional review board committee (IRB) at Tanta University, Faculty of Medicine (IRB number 35698/9/22). Ethical approvals were also obtained from Egypt, Algeria, India, Iraq, Pakistan, Palestine, Libya, Saudi Arabia, Sudan, Syria, and Yemen.

### Data analysis

The data were organized in a Microsoft Excel sheet and then imported and analyzed using R Statistical Software (v4.1.3; R Core Team 2022). Frequencies and percentages were used to describe the categorical variables for baseline demographic characteristics. Regarding knowledge level, the knowledge questions have been recorded as 1 for the correct answer and 0 for the incorrect one. Students who obtained a score of ≥ 70% were deemed to have a high level of knowledge, whereas those with a score of < 70% were classified as having a low level of knowledge concerning blood donation^8,14,21^. The results of the attitude and practice sections were presented as frequencies and percentages. The Chi-square test assessed the significant association between demographic characteristics, knowledge level, attitude toward blood donation, and blood donation practices. We employed univariate and multivariate regression analyses to identify predictors influencing knowledge level and donation behaviors, quantifying associations through odds ratios (OR) and adjusted odds ratios (aOR) with 95% confidence intervals (CI). A *p*-value of ≤ 0.05 was considered significant.

### Ethics approval and consent to participate

This survey was voluntary, and participants provided their informed consent by marking a checkbox to signify their willingness to take part in the study. Participants’ anonymity and confidentiality were ensured throughout the study, including data collection and analysis. Initial ethical approval was obtained from the institutional review board committee (IRB) at Tanta University, Faculty of Medicine (IRB number 35698/9/22). Ethical approvals were also obtained from the participating countries.

## Results

The study invited 14,625 individuals to complete the questionnaire, with 800 individuals either not eligible or declining participation. Therefore, 13,825 participants completed the questionnaire, with 5100 and 8725 using the English and Arabic forms, respectively. Data from 1219 participants were excluded from the analysis due to inconsistency, and the final analysis included 12,606 participants from 16 countries.

### Demographic characteristics

Egypt had the highest response rate of 10% among the 16 countries, while Palestine had the lowest response rate of 4.8%. Among the study participants, 36% were aged ≤ 20 years (n = 4543), while the over-20 age group constituted 64% (n = 8063). Furthermore, 7966 participants (63.2%) identified as females, and 10,091 participants (80%) were residents of urban areas. Most participants were enrolled in governmental universities (71.4%) and health science colleges (67%). About 90.3% did not have a history of congenital or chronic disease. Further details regarding demographic characteristics are presented in Table 1 and Online Appendix S1.

**Table 1:** Demographics and characteristics of the participants.

Variable N (%)	Health science college (N = 8452)	Non-health science college (N = 4154)	Total (N = 12,606)	*p*-value *
Age				< 0.001
≤ 20 years	2940 (34.8)	1603 (38.6)	4543 (36)	
> 20 years	5512 (65.2)	2551 (61.4)	8063 (64)	
Gender				< 0.001
Male	2943 (34.8)	1697 (40.9)	4640 (36.8)	
Female	5509 (65.2)	2457 (59.1)	7966 (63.2)	
Country of Residence				< 0.001
Algeria	432 (5.1)	301 (7.2)	733 (5.8)	
Bahrain	335 (4.0)	443 (10.7)	778 (6.2)	
Egypt	946 (11.2)	310 (7.5)	1256 (10.0)	
Ghana	395 (4.7)	314 (7.6)	709 (5.6)	
India	536 (6.3)	298 (7.2)	834 (6.6)	
Iraq	570 (6.7)	153 (3.7)	723 (5.7)	
Jordan	522 (6.2)	273 (6.6)	795 (6.3)	
Libya	592 (7.0)	520 (12.5)	1112 (8.8)	
Morocco	339 (4.0)	285 (6.9)	624 (5.0)	
Pakistan	452 (5.3)	177 (4.3)	629 (5.0)	
Palestine	464 (5.5)	145 (3.5)	609 (4.8)	
Saudi Arabia	360 (4.3)	262 (6.3)	622 (4.9)	
Sudan	955 (11.3)	176 (4.2)	1131 (9.0)	
Syria	695 (8.2)	104 (2.5)	799 (6.3)	
Turkey	464 (5.5)	176 (4.2)	640 (5.1)	
Yemen	395 (4.7)	217 (5.2)	612 (4.9)	
Original Residence				0.714
Rural	1678 (19.9)	837 (20.1)	2515 (20.0)	
Urban	6774 (80.1)	3317 (79.9)	10091 (80.0)	
Type of University				< 0.001
Governmental	5855 (69.3)	3151 (75.9)	9006 (71.4)	
International	179 (2.1)	154 (3.7)	333 (2.6)	
Private	2418 (28.6)	849 (20.4)	3267 (25.9)	
History of congenital or chronic diseases				0.354
Yes	807 (9.5)	419 (10.1)	1226 (9.7)	
No	7645 (90.5)	3735 (89.9)	11380 (90.3)	

*Chi-square test.

### Knowledge regarding blood donation

A total of 9842 participants (78.1%) were aware of their blood type, while 9760 participants (77.4%) knew about the right to voluntary blood donation. Only 5507 participants (43.7%) knew the amount of blood taken during a single donation, and 4984 participants (39.5%) knew the minimum interval between two successive donations. Additionally, 70.1% and 39.1% of participants knew the minimum age and weight requirements for donation, respectively. About 89.8% of participants knew that donated blood is tested before being transfused, while 58.9% knew that not all individuals with diabetes or hypertension could donate. Only 42.2% knew that smokers are eligible to donate, while 57.9% knew that having a fever on the donation day disqualifies a person. Meanwhile, 71.9% knew that pregnant women are ineligible to donate, while 16.1% knew that women can donate during menstruation. The health science college group had significantly higher knowledge scores than the non-health science college group (*p*-value < 0.001). Overall, only 3588 participants (28.5%) demonstrated a high level of knowledge (≥ 70% of correct answers), with a significantly higher percentage in the health science college group (34.7%) compared to the non-health science college group (15.7%), with a *p*-value of < 0.001). See Table 2 for detailed knowledge section results.

**Table 2:** Knowledge level of the participants toward blood donation.

Variable N (%)	Health science college (N = 8452)	Non-health science college (N = 4154)	Total (N = 12,606)	*p*-value *
Do you know your blood group (type)?				< 0.001
*Yes*	6857 (81.1)	2985 (71.9)	9842 (78.1)	
No	1595 (18.9)	1169 (28.1)	2764 (21.9)	
Are you aware of the right to voluntary blood donation?				< 0.001
*Yes*	6778 (80.2)	2982 (71.8)	9760 (77.4)	
No	1674 (19.8)	1172 (28.2)	2846 (22.6)	
What is the amount of blood taken in a single blood donation process?				< 0.001
*500 ml*	4411 (52.2)	1096 (26.4)	5507 (43.7)	
750 ml	359 (4.2)	224 (5.4)	583 (4.6)	
1000 ml	499 (5.9)	237 (5.7)	736 (5.8)	
I do not know	3183 (37.7)	2597 (62.5)	5780 (45.9)	
What is the minimum age limit for blood donation?				< 0.001
*18 years*	6211 (73.5)	2627 (63.2)	8838 (70.1)	
20 years	239 (2.8)	161 (3.9)	400 (3.2)	
21 years	121 (1.4)	91 (2.2)	212 (1.7)	
I do not know	1881 (22.3)	1275 (30.7)	3156 (25.0)	
What is the minimum weight limit for blood donation?				< 0.001
*50 kg*	3664 (43.4)	1262 (30.4)	4926 (39.1)	
60 kg	1217 (14.4)	560 (13.5)	1777 (14.1)	
70 kg	235 (2.8)	137 (3.3)	372 (3.0)	
I do not know	3336 (39.5)	2195 (52.8)	5531 (43.9)	
What minimum interval should be between two successive blood donation processes?				< 0.001
1 month	688 (8.1)	387 (9.3)	1075 (8.5)	
*3 months*	3793 (44.9)	1191 (28.7)	4984 (39.5)	
6 months	1579 (18.7)	726 (17.5)	2305 (18.3)	
I do not know	2392 (28.3)	1850 (44.5)	4242 (33.7)	
Will the donated blood be tested before transfusion into other persons?				< 0.001
*Yes*	7868 (93.1)	3450 (83.1)	11,318 (89.8)	
No	179 (2.1)	114 (2.7)	293 (2.3)	
I do not know	405 (4.8)	590 (14.2)	995 (7.9)	
Can all persons with diabetes or hypertension donate?				< 0.001
Yes	1192 (14.1)	425 (10.2)	1617 (12.8)	
*No*	5144 (60.9)	2284 (55.0)	7428 (58.9)	
I do not know	2116 (25.0)	1445 (34.8)	3561 (28.2)	
Can smokers donate?				< 0.001
*Yes*	3841 (45.4)	1477 (35.6)	5318 (42.2)	
No	2505 (29.6)	1373 (33.1)	3878 (30.8)	
I do not know	2106 (24.9)	1304 (31.4)	3410 (27.1)	
If a person has a fever on the donation day, should he or she donate?				< 0.001
Yes	607 (7.2)	356 (8.6)	963 (7.6)	
*No*	5345 (63.2)	1952 (47.0)	7297 (57.9)	
I do not know	2500 (29.6)	1846 (44.4)	4346 (34.5)	
Can a pregnant woman donate?				< 0.001
Yes	333 (3.9)	154 (3.7)	487 (3.9)	
*No*	6320 (74.8)	2741 (66.0)	9061 (71.9)	
I do not know	1799 (21.3)	1259 (30.3)	3058 (24.3)	
Can women donate during menstruation?				< 0.001
*Yes*	1513 (17.9)	511 (12.3)	2024 (16.1)	
No	4384 (51.9)	1739 (41.9)	6123 (48.6)	
I do not know	2555 (30.2)	1904 (45.8)	4459 (35.4)	
Overall knowledge level				< 0.001
High	2934 (34.7)	654 (15.7)	3588 (28.5)	
Low	5518 (65.3)	3500 (84.3)	9018 (71.5%)	

* Chi-square test; The italic answers are the correct answers; a high knowledge level is a score of ≥ 70% of the correct answers; a low knowledge level is a score of < 70% of the correct answers.

### Attitude toward blood donation

Approximately 24.2% of the participants reported receiving lectures or courses about donation, while 75% expressed their desire to receive training on blood donation. Nearly 90% of the participants reported being ready to donate blood if there was a serious shortage in the blood banks, and 79.6% encouraged nearby people to donate. Around 80% of the participants were willing to participate in any campaign organized by their university; moreover, 77.9% expressed their willingness to take responsibility for spreading accurate information about blood donation to the public. Concerning social media and blood donation calls, Facebook (26.8%), followed by Instagram (13.7%) and WhatsApp (10%), were the most commonly used platforms. Noteworthy, 43.5% of the participants reported not seeing any calls for blood donation on social media.

Additionally, 47.6% of the participants reported a positive attitude toward their friends toward blood donation, while 42.2% and 41.4% reported a positive attitude toward their universities and families, respectively. We observed significant differences in all items of the attitude section between students enrolled in health and non-health science colleges. Specifically, more students in health science colleges demonstrated a positive attitude toward blood donation and reported seeing calls for donation on social media. In addition, more students in health science colleges reported positive attitudes towards their friends and universities. Conversely, the positive attitude towards blood donation from families was more prominent in the non-health science colleges group. All of these differences were found to be statistically significant (*p*-value < 0.001), except for the difference in willingness to donate if the university organizes a donation campaign (*p*-value = 0.5). Furthermore, students with a high level of knowledge were found to have a significantly more positive attitude towards blood donation than those with a low level of knowledge. The detailed results of the attitude section can be found in Tables 3 and 4.

**Table 3:** Attitude of students of health and non-health science colleges toward blood donation.

Variable N (%)	Health science college (N = 8452)	Non-health science college (N = 4154)	Total (N = 12,606)	*p*-value *
Have you ever had courses or lectures on blood donation and its importance?				< 0.001
Yes	2454 (29.0)	595 (14.3)	3049 (24.2)	
No	5998 (71.0)	3559 (85.7)	9557 (75.8)	
Would you like to receive training on blood donation?				< 0.001
Yes	6667 (78.9)	2789 (67.1)	9456 (75.0)	
No	1785 (21.1)	1365 (32.9)	3150 (25.0)	
Would you donate if there is a serious shortage in blood banks?				< 0.001
Yes	7711 (91.2)	3676 (88.5)	11,387 (90.3)	
No	741 (8.8)	478 (11.5)	1219 (9.7)	
Do you encourage people around you to donate?				< 0.001
Yes	6878 (81.4)	3156 (76.0)	10,034 (79.6)	
No	1574 (18.6)	998 (24.0)	2572 (20.4)	
If your university organizes a donation campaign, would you participate and donate?				0.503
Yes	6759 (80.0)	3300 (79.4)	10,059 (79.8)	
No	1693 (20.0)	854 (20.6)	2547 (20.2)	
Would you like to take responsibility for spreading accurate information about blood donation to lay people?				< 0.001
Yes	6883 (81.4)	2935 (70.7)	9818 (77.9)	
No	1569 (18.6)	1219 (29.3)	2788 (22.1)	
If you have seen calls for donation on social media, name the most involved platform				< 0.001
Facebook	2454 (29.0)	922 (22.2)	3376 (26.8)	
Instagram	1119 (13.2)	604 (14.5)	1723 (13.7)	
LinkedIn	18 (0.2)	11 (0.3)	29 (0.2)	
Snapchat	13 (0.2)	23 (0.6)	36 (0.3)	
Telegram	29 (0.3)	2 (0.0)	31 (0.2)	
TikTok	28 (0.3)	22 (0.5)	50 (0.4)	
Twitter	248 (2.9)	153 (3.7)	401 (3.2)	
WhatsApp	915 (10.8)	351 (8.4)	1266 (10.0)	
YouTube	139 (1.6)	72 (1.7)	211 (1.7)	
I have not seen	3489 (41.3)	1994 (48.0)	5483 (43.5)	
Describe the attitude of your college/university toward blood donation				< 0.001
Very discouraging	984 (11.6)	797 (19.2)	1781 (14.1)	
Discouraging	945 (11.2)	515 (12.4)	1460 (11.6)	
Neutral	2650 (31.4)	1395 (33.6)	4045 (32.1)	
Encouraging	1765 (20.9)	651 (15.7)	2416 (19.2)	
Very encouraging	2108 (24.9)	796 (19.2)	2904 (23.0)	
Describe the attitude of your family toward blood donation				< 0.001
Very discouraging	952 (11.3)	389 (9.4)	1341 (10.6)	
Discouraging	1277 (15.1)	518 (12.5)	1795 (14.2)	
Neutral	2903 (34.3)	1352 (32.5)	4255 (33.8)	
Encouraging	1588 (18.8)	847 (20.4)	2435 (19.3)	
Very encouraging	1732 (20.5)	1048 (25.2)	2780 (22.1)	
Describe the attitude of your friends toward blood donation?				0.001
Very discouraging	667 (7.9)	421 (10.1)	1088 (8.6)	
Discouraging	915 (10.8)	452 (10.9)	1367 (10.8)	
Neutral	2809 (33.2)	1345 (32.4)	4154 (33.0)	
Encouraging	1984 (23.5)	912 (22.0)	2896 (23.0)	
Very encouraging	2077 (24.6)	1024 (24.7)	3101 (24.6)	

*Chi-square test.

**Table 4:** Attitude of students with high versus low levels of knowledge regarding blood donation.

Variable N (%)	High knowledge (N = 3588)	Low knowledge (N = 9018)	Total (N = 12,606)	*p*-value*
Have you ever had courses or lectures on blood donation and its importance?				< 0.001
Yes	1424 (39.7)	1625 (18.0)	3049 (24.2)	
No	2164 (60.3)	7393 (82.0)	9557 (75.8)	
Would you like to receive training on blood donation?				0.018
Yes	2744 (76.5)	6712 (74.4)	9456 (75.0)	
No	844 (23.5)	2306 (25.6)	3150 (25.0)	
Would you donate if there is a serious shortage in blood banks?				< 0.001
Yes	3333 (92.9)	8054 (89.3)	11,387 (90.3)	
No	255 (7.1)	964 (10.7)	1219 (9.7)	
Do you encourage people around you to donate?				< 0.001
Yes	3090 (86.1)	6944 (77.0)	10,034 (79.6)	
No	498 (13.9)	2074 (23.0)	2572 (20.4)	
If your university organizes a donation campaign, would you participate and donate?				< 0.001
Yes	2982 (83.1)	7077 (78.5)	10,059 (79.8)	
No	606 (16.9)	1941 (21.5)	2547 (20.2)	
Would you like to take responsibility for spreading accurate information about blood donation to lay people?				< 0.001
Yes	3010 (83.9)	6808 (75.5)	9818 (77.9)	
No	578 (16.1)	2210 (24.5)	2788 (22.1)	
If you have seen calls for donation on social media, name the most involved platform				< 0.001
Facebook	1126 (31.4)	2250 (25.0)	3376 (26.8)	
Instagram	590 (16.4)	1133 (12.6)	1723 (13.7)	
LinkedIn	7 (0.2)	22 (0.2)	29 (0.2)	
Snapchat	6 (0.2)	30 (0.3)	36 (0.3)	
Telegram	6 (0.2)	25 (0.3)	31 (0.2)	
TikTok	9 (0.3)	41 (0.5)	50 (0.4)	
Twitter	117 (3.3)	284 (3.1)	401 (3.2)	
WhatsApp	447 (12.5)	819 (9.1)	1266 (10.0)	
YouTube	69 (1.9)	142 (1.6)	211 (1.7)	
I have not seen	1211 (33.8)	4272 (47.4)	5483 (43.5)	
Describe the attitude of your college university towards blood donation				< 0.001
Very discouraging	316 (8.8)	1465 (16.2)	1781 (14.1)	
Discouraging	353 (9.8)	1107 (12.3)	1460 (11.6)	
Neutral	1067 (29.7)	2978 (33.0)	4045 (32.1)	
Encouraging	805 (22.4)	1611 (17.9)	2416 (19.2)	
Very encouraging	1047 (29.2)	1857 (20.6)	2904 (23.0)	
Describe the attitude of your family toward blood donation				< 0.001
Very discouraging	318 (8.9)	1023 (11.3)	1341 (10.6)	
Discouraging	454 (12.7)	1341 (14.9)	1795 (14.2)	
Neutral	1114 (31.0)	3141 (34.8)	4255 (33.8)	
Encouraging	752 (21.0)	1683 (18.7)	2435 (19.3)	
Very encouraging	950 (26.5)	1830 (20.3)	2780 (22.1)	
Describe the attitude of your friends toward blood donation				< 0.001
Very discouraging	211 (5.9)	877 (9.7)	1088 (8.6)	
Discouraging	326 (9.1)	1041 (11.5)	1367 (10.8)	
Neutral	1061 (29.6)	3093 (34.3)	4154 (33.0)	
Encouraging	941 (26.2)	1955 (21.7)	2896 (23.0)	
Very encouraging	1049 (29.2)	2052 (22.8)	3101 (24.6)	

*Chi-square test.

### Blood donation practice

Regarding blood donation practice, only 22.7% of the participants had donated before, and 55% of them donated irregularly. A small fraction of the participants (18.1%) had engaged in voluntary blood donation, and only 11.6% had donated once. Most participants (85.4%) expressed their intention to donate blood. We compared the practices of health and non-health science college students and found no significant difference in the donor ratio (*p*-value = 0.81). However, the differences in the frequency and type of donation were significant (*p*-value = 0.022, 0.043, respectively). We also observed significant differences in all aspects of donation practice between students with high and low knowledge (*p*-value < 0.001). Specifically, 34.6% of students with high knowledge had donated before, compared to only 17.9% of those with low knowledge. Moreover, 28.5% of the high-knowledge group, compared to only 13.9% of the low-knowledge group, practiced voluntary donation. Finally, 12.2% of the high-knowledge group had donated more than twice, compared to only 3.3% of the low-knowledge group. The detailed results of the practice section are presented in Tables 5 and 6.

**Table 5:** Practice of blood donation among health and non-health science colleges.

Variable N (%)	Health science college (N = 8452)	Non-health science college (N = 4154)	Total (N = 12,606)	*p*-value*
Have you ever donated?				0.811
Yes	1922 (22.7)	936 (22.5)	2858 (22.7)	
No	6530 (77.3)	3218 (77.5)	9748 (77.3)	
What type of blood donation have you practiced?				0.043
Voluntary (gives the blood of his or her own free will and receives no payment)	1561 (18.5)	718 (17.3)	2279 (18.1)	
Replacement (gives blood when it is required by a family or community member)	350 (4.1)	210 (5.1)	560 (4.4)	
Paid (received payment to donate)	11 (0.1)	8 (0.2)	19 (0.2)	
Never practiced	6530 (77.3)	3218 (77.5)	9748 (77.3)	
How many times have you donated?				0.122
Once	993 (11.7)	474 (11.4)	1467 (11.6)	
Twice	419 (5.0)	239 (5.8)	658 (5.2)	
More than twice	510 (6.0)	223 (5.4)	733 (5.8)	
Never donated	6530 (77.3)	3218 (77.5)	9748 (77.3)	
How often do you donate?	(N = 1922)	(N = 936)		0.022
Regularly, once a year	117 (6.1)	74 (7.9)	191 (6.7)	
Regularly, twice a year	137 (7.1)	50 (5.3)	187 (6.5)	
Regularly more than twice a year	73 (3.8)	41 (4.4)	114 (4.0)	
I donate but not regularly	1083 (56.3)	489 (52.2)	1572 (55.0)	
Only when someone close needs	512 (26.6)	282 (30.1)	794 (27.8)	
Do you intend to donate in the future?				0.744
Yes, I do	7229 (85.5)	3538 (85.2)	10,767 (85.4)	
No, I do not intend to	908 (10.7)	450 (10.8)	1358 (10.8)	
No, I am no longer medically eligible to donate	315 (3.7)	166 (4.0)	481 (3.8)	

*Chi-square test.

**Table 6:** Practice of blood donation among students with high vs low levels of knowledge regarding blood donation.

Variable N (%)	High knowledge (N = 3588)	Low knowledge (N = 9018)	Total (N = 12,606)	*p*-value*
Have you ever donated?				< 0.001
Yes	1243 (34.6)	1615 (17.9)	2858 (22.7)	
No	2345 (65.4)	7403 (82.1)	9748 (77.3)	
What type of blood donation have you practiced?				< 0.001
Voluntary (gives the blood of his or her own free will and receives no payment)	1024 (28.5)	1255 (13.9)	2279 (18.1)	
Replacement (gives blood when it is required by a family or community member)	217 (6.0)	343 (3.8)	560 (4.4)	
Paid (received payment to donate)	2 (0.1)	17 (0.2)	19 (0.2)	
Never practiced	2345 (65.4)	7403 (82.1)	9748 (77.3)	
How many times have you donated?				< 0.001
Once	533 (14.9)	934 (10.4)	1467 (11.6)	
Twice	271 (7.6)	387 (4.3)	658 (5.2)	
More than twice	439 (12.2)	294 (3.3)	733 (5.8)	
Never donated	2345 (65.4)	7403 (82.1)	9748 (77.3)	
How often do you donate?	(N = 1243)	(N = 1615)		< 0.001
Regularly, once a year	76 (6.1)	115 (7.1)	191 (6.7)	
Regularly, twice a year	121 (9.7)	66 (4.1)	187 (6.5)	
Regularly more than twice a year	67 (5.4)	47 (2.9)	114 (4.0)	
I donate but not regularly	707 (56.9)	865 (53.6)	1572 (55.0)	
Only when someone close needs	272 (21.9)	522 (32.3)	794 (27.8)	
Do you intend to donate in the future?				< 0.001
Yes, I do	3156 (88.0)	7611 (84.4)	10,767 (85.4)	
No, I do not intend to	283 (7.9)	1075 (11.9)	1358 (10.8)	
No, I am no longer medically eligible to donate	149 (4.2)	332 (3.7)	481 (3.8)	

*Chi-square test.

### Characteristics of blood donors and non-donors

We observed a significant difference in the distribution of participants based on their blood donation status. Those who had donated at least once were classified as donors. A majority of the donors were males (65.1%), whereas most of the non-donors were females (71.5%). In terms of knowledge regarding blood donation, approximately 43% of the donors had a high level of knowledge compared to only 24.1% of the non-donors (*p*-value < 0.001). However, the two groups had no significant difference based on college type (health or non-health science college), with a *p*-value of 0.8. We provided the details of the characteristics of blood donors and non-donors in Online Appendix S2.

### Factors that motivate and hinder blood donation

Regarding motivating factors, most participants (66%) were motivated to donate due to a friend or family member in need, followed by public promotion (42%). While 39% of participants were motivated by the potential health benefits of donating, only 13% felt that a national disaster would motivate them to donate. The least motivating factor was religious belief, which only motivated 1% of participants. Regarding barriers to donation, 37% of participants reported not donating because no one had asked. Other reasons included medical ineligibility (33%), fear of pain, bleeding, or infection (18%), concerns that donation would negatively affect their health (18%), difficulty accessing donation centers (15%), and medical mistrust (14%). Lack of time was the least cited barrier to donation (0.3%). More detailed information on motivating and preventing factors can be found in Online Appendix S3 and Online Appendix S4.

### Factors that influence blood donation knowledge

As indicated by the multivariate analysis, individuals aged > 20 years demonstrated notably higher odds of having a high level of knowledge compared to those aged ≤ 20 years (aOR 1.77, 95% CI 1.62–1.93, *p* < 0.001). Additionally, students enrolled in private and international universities had higher odds of having a high level of knowledge compared to those in governmental universities (aOR: 1.19; 95% CI 1.09–1.30; *p*-value < 0.001 and aOR: 1.44; 95% CI 1.13–1.84; *p*-value = 0.003, respectively). In contrast, students in non-health science colleges had significantly lower odds of having a high level of knowledge than students in health science colleges (aOR: 0.36; 95% CI 0.32–0.39; *p*-value < 0.001); refer to Table 7 for details.

**Table 7:** Univariate and multivariate logistic regression analysis showing predictors of knowledge level among the study participants.

Dependent: Knowledge level	Low knowledge (N = 9018)	High knowledge (N = 3588)	Univariate OR (95% CI ^a^	Multivariate aOR (95% CI)
*Age*				
≤ 20 years	3582 (78.8)	961 (21.2)	–	–
> 20 years	5436 (67.4)	2627 (32.6)	1.80 (1.65–1.96, *p* < 0.001)	1.77 (1.62–1.93, *p* < 0.001)
*Gender*				
Male	3297 (71.1)	1343 (28.9)	–	–
Female	5721 (71.8)	2245 (28.2)	0.96 (0.89–1.04, *p* = 0.361)	0.96 (0.89–1.05, *p* = 0.386)
*Original residence*				
Rural	1785 (71.0)	730 (29.0)	–	–
Urban	7233 (71.7)	2858 (28.3)	0.97 (0.88–1.06, *p* = 0.484)	0.93 (0.85–1.03, *p* = 0.187)
*Type of University*				
Governmental	6584 (73.1)	2422 (26.9)	–	–
International	229 (68.8)	104 (31.2)	1.23 (0.97–1.56, *p* = 0.081)	1.44 (1.13–1.84, *p* = 0.003)
Private	2205 (67.5)	1062 (32.5)	1.31 (1.20–1.43, *p* < 0.001)	1.19 (1.09–1.30, *p* < 0.001)
*College*				
Health science college	5518 (65.3)	2934 (34.7)	–	–
Non-health science college	3500 (84.3)	654 (15.7)	0.35 (0.32–0.39, *p* < 0.001)	0.36 (0.32–0.39, *p* < 0.001)
*History of congenital or chronic diseases*				
No, I don’t	8122 (71.4)	3258 (28.6)	–	–
Yes I have	896 (73.1)	330 (26.9)	0.92 (0.80–1.05, *p* = 0.207)	0.93 (0.81–1.06, *p* = 0.287)

^**a**^*OR* Odds ratio, *aOR* Adjusted odds ratio, *CI* Confidence interval.

### Factors that influence blood donation status

Investigating predictors of blood donation showed that participants aged > 20 years were significantly more likely to be blood donors compared to those ≤ 20 years, with aOR of 2.21 (95% CI 1.99–2.45, *p* < 0.001). However, being female significantly reduced the odds of being a blood donor (aOR: 0.21; 95% CI 0.19–0.23; *p*-value < 0.001). Urban residence was associated with lower odds of donation status than rural residence in the univariate analysis (OR: 0.83, 95% CI 0.75–0.92, *p* < 0.001). However, this association became non-significant in the multivariate analysis (aOR: 0.90, with a *p*-value of 0.061). Participants with a history of congenital or chronic diseases were less likely to have a positive donation status (aOR: 0.81, 95% CI 0.68–0.96, *p* = 0.014). In addition, those having a low knowledge level had significantly lower odds of blood donation compared to those with high knowledge levels (aOR: 0.40, 95% CI 0.37–0.44, *p* < 0.001), Table 8.

**Table 8:** Univariate and multivariate logistic regression analysis showing predictors of donation status.

Dependent: Donation status	No (N = 9748)	Yes (N = 2858)	Univariate OR (95% CI)^a^	Multivariate aOR (95% CI)
*Age*				
≤ 20 years	3948 (86.9)	595 (13.1)	–	–
20 years	5800 (71.9)	2263 (28.1)	2.59 (2.35–2.86, *p* < 0.001)	2.21 (1.99–2.45, *p* < 0.001)
*Gender*				
Male	2780 (59.9)	1860 (40.1)	–	–
Female	6968 (87.5)	998 (12.5)	0.21 (0.20–0.23, *p* < 0.001)	0.21 (0.19–0.23, *p* < 0.001)
*Original residence*				
Rural	1879 (74.7)	636 (25.3)	–	–
Urban	7869 (78.0)	2222 (22.0)	0.83 (0.75–0.92, *p* < 0.001)	0.90 (0.81–1.01, *p* = 0.061)
*History of congenital or chronic diseases*				
No, I don’t	8727 (76.7)	2653 (23.3)	–	–
Yes I have	1021 (83.3)	205 (16.7)	0.66 (0.56–0.77, *p* < 0.001)	0.81 (0.68–0.96, *p* = 0.014)
*Knowledge level*				
High	2345 (65.4)	1243 (34.6)	–	–
Low	7403 (82.1)	1615 (17.9)	0.41 (0.38–0.45, *p* < 0.001)	0.40 (0.37–0.44, *p* < 0.001)

^**a**^*OR* Odds ratio, *aOR* Adjusted odds ratio, *CI* Confidence interval.

## Discussion

We conducted a cross-sectional study using a self-administered survey to assess the knowledge, attitude, practice, motivators, and barriers to blood donation among 12606 university students from 16 countries. This sample size is much larger than previous similar studies^1–3,8,10,12–15,17,19,22–27^. University students are an essential population to investigate as potential blood donors. Our findings revealed a low level of knowledge and practice of blood donation among the participants, although they held a positive attitude toward it.

### Knowledge level

Our study revealed that a low percentage (28.5%) of university students have good knowledge of blood donation, which is similar to studies from Spain and Portugal (30%)^1^ but higher than in Iran (15.5%)^27^. In contrast, studies from India (57%)^17^ and Saudi Arabia (60.2%)^14^ reported higher knowledge levels. Nevertheless, a recent study from Saudi Arabia showed that only 3% of students had a high knowledge level, while 44.4% had moderate knowledge^8^. About 78.1% of our participants knew their blood type, similar to a study in Brazil (79.1%)^19^, while a higher percentage of students in Qatar knew such essential and critical information^13^.

Furthermore, our results showed that higher age was associated with a high knowledge level, which is consistent with previous studies^13,17^. This may be explained by the fact that individuals’ general knowledge and awareness increase with age. Additionally, students in their first year of university tend to be burdened with between, which could limit their ability to acquire additional knowledge. However, an Ethiopian study did not find a significant association between age and knowledge level regarding blood donation^3^.

Females also had higher knowledge levels compared to males, which is commonly found in previous studies^17,22,27^. According to an Ethiopian study, the correlation between gender and knowledge level regarding donation is prominent in health science students^3^. However, the study found no significant relationship between gender and knowledge level among non-health science students. Likewise, our study did not detect any significant association between gender and knowledge level among all university students. In contrast, a study conducted in Saudi Arabia showed that males had significantly higher levels of knowledge^8^.

The study found that students in health science colleges had significantly higher levels of knowledge (34.7%) than those in non-health science colleges (15.7%). This pattern is consistent with a study conducted in Ethiopia, where only 13.9% of non-health science students had good or adequate knowledge, while 79.4% of health science students had a good knowledge level^3^. A similar result was also reported in India^17^. These disparities in knowledge levels may be attributed to variable socioeconomic and cultural backgrounds and differences in the distribution of colleges in each study.

A majority of the participants in our study exhibited positive attitudes toward blood donation. Similar positive responses toward blood donation were reported in studies conducted in Saudi Arabia, Pakistan, and India^14,17^. In our study, 24.2% of participants had received education on blood donation, whereas around 30% of participants in a study conducted in Saudi Arabia had attended lectures on this topic^14^. These findings emphasize the necessity of organizing effective campaigns to encourage blood donation, particularly at the university and college levels. Our study indicated that 79.8% of participants would be willing to donate if the university organized such campaigns, while a study conducted in Saudi Arabia revealed that 84% of their participants expressed a similar willingness to donate^14^. Furthermore, the results of our study and the Saudi Arabian study^14^ suggest that greater attention should be given to social and public media, as around 43.5% and 41% of our participants and Saudi participants, respectively, reported not having seen any calls for blood donation on these platforms.

### The practice of blood donation

According to our findings, only 22.7% of participants had donated blood at least once. This rate is higher than what has been reported in Iran (10%) and Qatar (15%)^13,27^. However, other studies have shown a higher percentage of students with a history of blood donation, including Greece (24%)^23^, Ethiopia (27.2%)^3^, Spain (28.1%)^24^, Saudi Arabia (29%)^8^, Brazil (32.6%)^19^, Italy (34%)^12^, Canada (43.8%)^3^, China (50%)^2^, India (55%)^17^, and the United States (56%)^28^. In our study, most donors donated blood voluntarily, which is consistent with previous studies in Greece^23^ and Saudi Arabia^14^.

Our findings suggest that age is significantly associated with blood donation status, with an increase in age resulting in a higher likelihood of donating blood. These results are consistent with previous research demonstrating that higher ages positively correlate with blood donation^2,8,13,17^. We observed that more donors (34.6%) had higher knowledge levels than those with low or inadequate knowledge levels (17.9%). Therefore, having a high knowledge level increases the likelihood of blood donation. Other studies have similarly found that donors tend to have higher knowledge levels than non-donors^2,8,12^. In contrast, a study conducted in Ethiopia found no significant association between knowledge level and donation status^3^. Interestingly, a study in India^17^ reported that inadequate knowledge increased the odds of donation among their sample, which contradicts our findings. This discrepancy highlights the need for standardization and validation of information provided through initiatives, media, and educational curricula in schools and universities.

Our findings suggest that a significant association exists between gender and blood donation status, with a higher proportion of males (40.1%) being donors compared to females (12.5%). This gender disparity in blood donation is well-documented in studies from various countries^3,10,13,17,22,23,27^. In addition to previous research, our findings suggest that males are more likely to donate blood despite females having a higher knowledge level, indicating that knowledge is not always the sole factor influencing donation behavior. This trend may be partially explained by the fact that more women in low- and middle-income countries suffer from anemia, which can disqualify them from donating^17,25^. Additionally, cultural taboos affecting women can be a barrier to donation, although this may be less prevalent among university students due to their relatively high socioeconomic and educational status^10^. A systematic review identified that weight requirements and adverse effects such as dizziness could discourage women from donating, despite being more altruistic than men^29^. Therefore, females represent a significant potential pool of donors in developing countries, and addressing barriers to donation and improving their health status could increase participation in blood donation initiatives.

Our study found no significant association between the field of study (health vs. non-health) and blood donation, as the percentage of donors did not differ significantly between health and non-health science students, despite the significant difference in knowledge level. This finding is consistent with other studies showing that good knowledge does not always translate into donation behavior^8^. However, a study of young adults in Hong Kong, China, found that studying in health science or medical fields significantly increased the likelihood of donation^2^. Surprisingly, studies in India and Pakistan found that donation was more common among students in non-medical fields^10,17^. These results highlight the need to explore other factors that may encourage or discourage students from donating blood.

Although there were significant differences in knowledge levels among the different types of universities, our study found no significant difference in blood donation practice. This suggests that having good knowledge does not necessarily lead to good practice, and other factors may be at play. To our knowledge, this is the first study to examine the association between types of universities and blood donation practice or knowledge, and further research is needed to explore potential explanations for our findings.

### Motivators for and barriers against donation

66% of participants would be willing to donate blood if a friend or family member needed it, while 44% would do so for public recognition. Helping others or altruism was the most frequently reported motivation for donation, which is consistent with studies from various regions^9,13,19,29^. Personal health benefit was the most commonly reported motivation for donation, with 39% of our population indicating they would donate because it is healthy for the donor. This may be attributed to insufficient awareness among the participants, most of whom were in their first or second year of study^22^. This highlights the need to increase altruistic behavior among university students, especially those young or in their first year.

In our study, the most frequently reported reason for not donating was “no one asked,” with 37% of participants citing this as a factor. Previous studies^10,13,17^ have similarly found that the lack of opportunity, including not being asked, and fear of potential adverse effects during or after donation are common reasons for not donating. Additionally, 18% of our participants reported that fear of pain, infection, or other health complications after donation prevented them from donating. Another frequently reported reason among non-donors was the belief that they were medically ineligible or unfit for donation, which accounted for 33% of responses in our study. This could be partly due to insufficient knowledge about the health requirements for donation^10^. This reason was also the most commonly reported among non-donors in studies conducted in Saudi Arabia, Brazil, and Greece^8,19,23^.

While some previous studies have shown that the availability of mobile donation centers is a motivating factor for donation^13^, our study found that 15% of our student population has not donated due to the lack of accessible donation centers. This can increase the number of donors by providing more donation centers in convenient locations for these students. Medical mistrust was identified as a significant barrier to voluntary activities that involve direct contact with healthcare workers and policies^30^, and this was the case for approximately 14% of our participants. Notably, our study only included university students, which provides insight into the level of trust in healthcare institutions and policies in our region. This issue is particularly relevant for minority groups in each country and countries with low-quality healthcare systems^31^. This phenomenon may also be linked to the substandard level of governmental or public health services in numerous countries within the MENA region, indicating the urgent need for significant improvement^32,33^. We must be cautious when motivating students or the general population, as incentives or motivation without adequate awareness of the donation process and its health requirements may attract high-risk populations who may provide false information to receive the incentive. Additionally, extrinsic motivations may compromise the intrinsic motivations of donors, reducing their long-term desire to donate once the incentives are no longer available^11^.

### Strengths and limitations

This study represents the first large-scale multicenter investigation of its kind in our geographical region. Our study included 12,606 students from 16 countries, mainly from the MENA region. Moreover, our team of collaborators made tremendous efforts to ensure that a representative sample was collected in each country. Prior studies had much smaller sample sizes, often limited to one or two countries, and focused solely on health science students or medical students. In contrast, our study included non-health science college students and students from various types of universities, including governmental, private, and international universities. This comprehensive approach provides extensive and valuable insights into healthcare regulations and policies and paves the way for future targeted studies. Our study explored motivators for and barriers against blood donation, whereas previous studies only assessed motivators among donors and barriers among non-donors. This novel approach provides a complete understanding of the factors influencing blood donation. In addition, our study provides up-to-date information about blood donation in our region and fills a significant gap in the literature that aids in healthcare regulations and policies and paves the way for future targeted studies. University students represent a vital sector of our community. If their issues are appropriately addressed, voluntary donations may be a reliable source of blood supply instead of replacement donations.

However, it is important to note that our study has some limitations. Firstly, we utilized a cross-sectional design with convenience and snowball sampling, where the collaborators distributed a self-administered questionnaire on social media platforms. This data collection method may be susceptible to social desirability bias, as some students may have reported unreal information due to the socially desirable nature of blood donation. Selection bias may have also occurred, as only those who could respond to the online survey participated, even though we provided a printed (manual) version for those who could not fill in the online form. Additionally, our sample may have oversampled students with good backgrounds and attitudes towards donation while under-sampling those who do not know or care about our topic. Since our study only included university students, who may have higher social, economic, and educational backgrounds than the rest of the population in some countries, our results may not be generalizable over the entire population. The low response rate of non-health science students could have also affected our results. Moreover, since we did not imply a longitudinal design, we could not assess actual future intentions. While we have made efforts to collect comprehensive data from 16 countries, variations in regulations pertaining to weight, volume, and blood donation during menstruation may introduce limitations to the generalizability of our findings. Future research could delve deeper into these regulatory variations to strengthen the reliability and applicability of findings on a global scale.

Based on this, we emphasize that the generalization of the study results could primarily apply to the countries involved in this study. This limitation arises from potential racial and demographic disparities across nations, which may impact the transferability of findings to other regions. This underscores the need for caution when extrapolating findings to populations with distinct racial and demographic profiles.

## Recommendations

Additional interventions are required to improve knowledge and encourage blood donation among students. These initiatives should provide validated information on the importance, necessity, and requirements of donation and information about the actual rates of complications. Misconceptions, myths, and irrational fears should also be addressed. Health-science students can be crucial in increasing public awareness and promoting regular voluntary blood donation. They can effectively dispel social and cultural myths and unfounded fears^23^. Students with good knowledge and attitudes should be engaged in developing new and attractive recruitment methods. Public universities should incorporate essential community voluntary activities, such as blood donation, into their policies. Essential information about blood donation could also be incorporated into the curriculum of colleges, regardless of field or year of study^15^. More accessible or mobile donation centers should be provided in convenient locations for students who face transportation challenges, and extending the working hours of donation centers can be an option. Mobile applications for recruiting and retaining potential and previous blood donors could be an effective solution, but they may only be available in high-income countries with good infrastructure^13^. Social mobile applications such as WhatsApp could creatively bridge the gap between donors, blood banks, and patients needing blood^34^.

## Conclusions

Our study findings indicate that university students demonstrate a notable lack of knowledge about blood donation. Health science students, however, tend to exhibit higher levels of awareness than those studying in non-health science disciplines. Despite displaying generally positive attitudes towards blood donation, the actual donation rates among students are low, with negligible differences between health and non-health science students. Therefore, implementing targeted educational campaigns and improving accessibility to blood donation opportunities are imperative to cultivate a culture of blood donation within the university student population.

### Supplementary Information

Supplementary Information.

## Data Availability

All data relevant to the study are included in the article or uploaded as supplemental information.
