# Supplementary material for: Unveiling blood donation knowledge, attitude, and practices among 12,606 university students: a cross-sectional study across 16 countries
Source: Sci Rep. 2024 Apr 8;14:8219. doi: 10.1038/s41598-024-58284-4 (PMC11001850; doi:10.1038/s41598-024-58284-4)
Supplement: Supplementary file 1 — Supplementary Information. [file 41598_2024_58284_MOESM1_ESM.docx]

***Appendix S1: History of congenital or chronic diseases among the study participants***

| **Disease ^a^** | **Frequency (%)**  **(N = 1226)** |
| --- | --- |
| Blood diseases | 563 (46 %) |
| Cardiovascular diseases | 132 (11 %) |
| Asthma | 125 (10 %) |
| Diabetes Mellitus | 104 (8 %) |
| Gastrointestinal tract disorder | 65 (5 %) |
| Allergy | 55(4 %) |
| Kidney diseases | 41 (3 %) |
| Neurological condition | 39 (3 %) |
| Thyroid disorder | 33 (3 %) |
| Skin disease | 31 (3 %) |
| Eye condition | 26 (2 %) |
| Polycystic ovary syndrome | 25 (2 %) |
| Liver diseases | 19 (2 %) |
| Cancer | 18 (1 %) |
| Psychiatric condition | 14 (1 %) |
| Others **^b^** | 43 (4 %) |
| **^a^** Multiple responses  **^b^** Others: amyloidosis, endometriosis, musculoskeletal, autoimmune, ear, and congenital diseases | |

***Appendix S2*: *Donors and non-donors’ profile***

| **Variable N (%)** | **Donors**  **(N=2858)** | **Non-donors (N=9748)** | **Total (N=12606)** | **P-value** |
| --- | --- | --- | --- | --- |
| **Gender** |  |  |  | <0.001 |
| - Male | 1860 (65.1) | 2780 (28.5) | 4640 (36.8) |  |
| - Female | 998 (34.9) | 6968 (71.5) | 7966 (63.2) |  |
| **Original Residence** |  |  |  | 0.001 |
| - Rural | 636 (22.3) | 1879 (19.3) | 2515 (20.0) |  |
| - Urban | 2222 (77.7) | 7869 (80.7) | 10091 (80.0) |  |
| **Type of University** |  |  |  | 0.158 |
| - Governmental | 2007 (70.2) | 6999 (71.8) | 9006 (71.4) |  |
| - International | 86 (3.0) | 247 (2.5) | 333 (2.6) |  |
| - Private | 765 (26.8) | 2502 (25.7) | 3267 (25.9) |  |
| **College** |  |  |  | 0.811 |
| - Health science college | 1922 (67.2) | 6530 (67.0) | 8452 (67.0) |  |
| - Non-health science college | 936 (32.8) | 3218 (33.0) | 4154 (33.0) |  |
| **History of congenital or chronic diseases** |  |  |  | <0.001 |
| - No, I do not | 2653 (92.8) | 8727 (89.5) | 11380 (90.3) |  |
| - Yes, I have | 205 (7.2) | 1021 (10.5) | 1226 (9.7) |  |
| **Knowledge level** |  |  |  | <0.001 |
| - High | 1243 (43.5) | 2345 (24.1) | 3588 (28.5) |  |
| - Low | 1615 (56.5) | 7403 (75.9) | 9018 (71.5) |  |

***Appendix S3: Motivating factors for the study participants to donate***

| **Motivating factor(s) for donation? ^a^** | **Frequency (%)**  **(N = 12606)** |
| --- | --- |
| Family or friend is in need | 8259 (66 %) |
| Public promotion | 5238 (42 %) |
| It is healthy for me to donate | 4859 (39 %) |
| National disaster | 1610 (13 %) |
| Free health check | 1164 (9 %) |
| Extra marks and academic support | 493 (4 %) |
| Approved certificates | 393 (3 %) |
| Monetary reward | 345 (3 %) |
| A charitable contribution | 132 (1 %) |
| Religious motive | 94 (1 %) |
| Others **^b^** | 55 (0.4 %) |
| **^a^** Multiple responses  **^b^** Others: governmental issues, rewarding feeling after donation, life-threatening situations only, rare blood group, and easily accessible donation center | |

***Appendix S4: Barriers against blood donation among the university students***

| **What is/are the barrier(s) that prevent(s) you from the donation? ^a^** | **Frequency (%)**  **(N = 12606)** |
| --- | --- |
| No one asked | 4722 (37 %) |
| Medically not eligible | 4160 (33 %) |
| Fear of pain, bleeding, or infection | 2317 (18 %) |
| I think it would affect my health | 2306 (18 %) |
| Not accessible donation center | 1913 (15 %) |
| Medical mistrust | 1720 (14 %) |
| Objections from family members | 1155 (9 %) |
| Religious barrier | 194 (2 %) |
| No barrier | 118 (1 %) |
| No time or chance to donate | 39 (0.3 %) |
| Others | 81 (1 %) |
| **^a^** Multiple responses  **^b^** Others: procrastination, females and/or foreigners not allowed to donate, insufficient motive, never thought about it, unreasonable fear, fear of discovering a disease, not having excess blood, and the blood may go to the wrong person. | |
